# A Novel Electrocardiography Model for the Diagnosis of Acute Pulmonary Embolism

**DOI:** 10.3389/fcvm.2022.825561

**Published:** 2022-04-11

**Authors:** Xiao-Feng Su, Na Fan, Xue-Mei Yang, Jun-Mei Song, Qiong-Hui Peng, Xin Liu

**Affiliations:** ^1^Ultrasound in Cardiac Electrophysiology and Biomechanics Key Laboratory of Sichuan Province, Sichuan Provincial People's Hospital, University of Electronic Science and Technology of China, Chengdu, China; ^2^Department of Echocardiography and Non-Invasive Cardiology Laboratory, Chinese Academy of Sciences Sichuan Translational Medicine Research Hospital, Chengdu, China; ^3^Sichuan Provincial People's Hospital, Chengdu, China

**Keywords:** acute pulmonary embolism (APE), electrocardiography, clinical prediction model, SPPH-ECG model, test accuracy

## Abstract

Acute pulmonary embolism (acPE) is a severe disease that is often misdiagnosed as it is difficult to detect quickly and accurately. In this study, a novel electrocardiogram (ECG) model was used to estimate the probability of acPE rapidly *via* analysis of ECG characteristics. A total of 327 patients with acPE who were diagnosed at the Sichuan Provincial People's Hospital (SPPH) between 2018 and 2021 were retrospectively studied. A total of 331 patients were randomly selected as the control group, which included patients hospitalized during the same time period. The control group included patients who presented with characteristic symptoms of acPE, but this diagnosis was ruled out following further diagnostic testing. This study compared the diagnostic value of the ECG model with those of another ECG scoring model (Daniel-ECG score) and the most common prediction models (Wells score and Geneva score). This study established an ECG-predictive model using analysis of the ECG abnormalities in patients with acPE. The final ECG model included certain novel ECG signs that had not been incorporated in the previous ECG score of the patients, and thus, compared to the previous ECG score, exhibited a more favorable area under the receiver operating characteristic curve (AUC) value (0.8741). The model developed in this study was named the SPPH-ECG model. Furthermore, this study compared the SPPH-ECG model with Daniel-ECG score, Wells score, and Geneva score, and the SPPH-ECG model was demonstrated to exhibit a superior AUC value (0.8741), sensitivity (79.08%), negative predictive value (79.52%), and test accuracy (79.42%), while the Geneva score presented superior specificity (100%) and positive predictive value (100%) compared with the SPPH-ECG model. In conclusion, the SPPH-ECG model may play a role in ruling out acPE in patients during diagnostic testing and diagnose acPE rapidly and accurately in combination with the Geneva scoring system.

## Introduction

Acute pulmonary embolism (acPE) is a common cardiovascular disease that causes high patient morbidity and mortality. Rapid diagnosis of acPE can be challenging, and a high number of deaths due to this disease occur prior to diagnosis. Electrocardiogram (ECG) is one of the first tests to be performed in the emergency department in patients with cardiac or respiratory symptoms because it is quick to use, readily available, cost-effective, and exhibits no potential adverse effects (1). In clinical practice and when using the current European Society of Cardiology (ECE) acPE guidelines, the 12-lead ECG can be applied to estimate the pre-test probability of acPE, and the results can be assessed by clinical judgment, but this lacks standardization (2). Standardized prediction rules, such as the Wells and Geneva scores, can also be used and do not include the use of an ECG (2). While no isolated ECG abnormality has been definitively associated with acPE, certain constellations of ECG abnormalities have been indicated to be reasonably specific to this disease (3–6). ECG abnormalities in patients with acPE are being increasingly reported and characterized (7–12). Daniel et al. developed an ECG scoring system in 2001, whereby points were assigned for the ECG abnormalities, namely, sinus tachycardia (2 points), incomplete right bundle branch block (RBBB) (2 points), complete RBBB (3 points), T-wave inversion in leads V1–V4 (0–4 points), S wave in lead I (0 points), Q wave in lead III (1 point), inverted T in lead III (1 point), and entire S1Q3T3 complex (2 points) (6). The ECG prognostic score produced by the aforementioned scoring system aimed to provide a clinical tool that could be used to aid in the diagnosis of acPE. However, additional research into the ECG characteristics of patients with acPE indicated that a number of ECG abnormalities that exhibited valuable prognostic information were not included in this scoring criteria and included following abnormalities ST-segment depression (STD), ST-segment elevation (STE), qR/QR/Qr in lead V1, QRS fragmentation, atrial fibrillation, low-voltage QRS, axis deviation, P pulmonale, long QT (LQT), and S1S2S3 syndrome (13–16). The aim of this study was to create a novel ECG model that could be used to rapidly estimate the probability of acPE *via* the analysis of ECG characteristics in patients with acPE.

## Patients and Methods

### Patient Selection

In this retrospective study, all patients diagnosed with acPE exhibited a filling defect in the pulmonary artery, which was detected by computed tomography pulmonary angiography (CTPA). Furthermore, a 12-lead ECG was used to record data within 48 h from the onset of patient symptoms. In total, 327 patients (152 men and 175 women, mean age 65.25 ± 15.27 years) were enrolled in this study.

Additionally, a total of 331 patients (222 men and 109 women, mean age 61.73 ± 14.71 years) were enrolled randomly as the control cohort. These patients exhibited cardiopulmonary disease that was associated with symptoms characteristic to acPE (acute dyspnea, chest pain, hemoptysis, or syncope), but acPE was ruled out upon further examination. The exclusion criteria for all patients were previous cardiac dysfunction, complete or incomplete left bundle branch block (LBBB), serious primary pulmonary disease (serious pulmonary emphysema or pneumonitis), chronic thromboembolic pulmonary hypertension, prior pacemaker implantation, and nonavailability of relevant information in electronic medical charts.

### Data Acquisition

In a previous study, the following ECG findings, which have previously been indicated to be associated with acPE, were evaluated, namely, tachycardia (>100 beats/min), atrial arrhythmia, P pulmonale (P waves with amplitudes ≥2.5 mm in limb leads or >1.5 mm in lead V1), right-axis deviation (QRS electrical axis >90°), presence of the S1S2S3 pattern (presence of S waves with amplitudes ≥1.5 mm in leads I–III), presence of the S1Q3T3 pattern (presence of S waves in lead I and Q waves in lead III, each having amplitudes >1.5 mm; in association with a negative T wave in lead III), low voltage (overall deflection of QRS complex 5.0 mm in all limb leads), clockwise rotation (shift in the transition zone [R = S] in the precordial leads to V5 or beyond), an increased R wave of lead AVR (R/S ≥ 1), frequent atrial premature beats (atrial premature beats ≥3), STE ≥ 1.0 mm, and STD (depression of horizontal or downsloping ST segments ≥ 0.5 mm in the absence of complete bundle branch block or ventricular hypertrophy). ST segment deviation was measured manually at the J point and to the nearest 0.5 mm (6, 7, 17–21). QRS fragmentation in leads aVR and V1-3 and inferior leads were defined according to Das et al. (22) and Macfarlane et al. (23), respectively.

Relevant clinical history, blood test indicators, and echocardiography criterion data were collected in each patient. These data included information on recent surgeries or immobility, prior malignancy, D-dimer level, deep vein thrombosis (DVT), hemoptysis, and pulmonary hypertension. All factors assessed are presented in Table 1.

**Table 1:** The 27 ECG and 12 clinical characteristics analyzed in our study.

ECG1	TWI in leads V1–V3
ECG2	T wave inversion in lead V1 (0, <1 mm,1–2 mm,>2 mm)
ECG3	T wave inversion in lead V2 (0, <1 mm,1–2 mm,>2 mm)
ECG4	T wave inversion in lead V3 (0, <1 mm,1–2 mm,>2 mm)
ECG5	S1S2S3 pattern
ECG6	Heart rate
ECG7	STE in lead AVR (0 vs. 1)
	STD in lead AVR (0 vs. 2)
ECG8	qR/QR/Qr in lead V1
ECG9	STE in lead V1-V3 (0 vs. 1)
	STD in lead V1-V3 (0 vs. 2)
ECG10	STE in lead V1-V3(V1>V2>V3)
ECG11	Q wave in the inferior leads(Q>0.15 mv)
ECG12	Long QT
ECG13	Right bundle branch block (IRBBB 1, CRBBB 2)
ECG14	TWI in leads V1-V4
ECG15	Tachycardia
ECG16	Right axis deviation
ECG17	S1Q3T3
ECG18	Clockwise rotation
ECG19	Atrial fibrillation
ECG20	V1 R/S >1 or RV1≥1.0mv or RV1 + SV5≥1.2 mv
ECG21	P Pulmonale
ECG22	Frequent PAC
ECG23	STD in V4-V6
ECG24	STE in any lead
ECG25	STD in any lead
ECG26	Low QRS voltage
ECG27	R/S≥1 in lead AVR
SEX	SEX
AGE	AGE
History1	Surgery or fracture within the past month
History2	Previous PE or DVT
History3	Hemoptysis
History4	Active cancer
History5	HR≥100 bpm
History6	Unilateral lower-limb pain
History7	Pain on lower-limb deep venous palpation and unilateral edema
History8	Less likely the other disease
PA	Pulmonary hypertension
D2	D- dimer

*TWI, T-wave inversion; the S1S2S3 pattern, presence of S waves with amplitudes ≥1.5 mm in leads I–III; STE, ST-segment elevation; STD, ST-segment depression; the S1Q3T3 pattern, presence of S waves in lead I and Q waves in lead III, each having amplitudes >1.5 mm; in association with a negative T wave in lead III; CRBBB, complete right bundle branch block; IRBBB, incomplete right bundle branch block; frequent PAC: premature atrial contraction (PAC) ≥3 times in 10 s or PAC ≥ 5 times in 1 min*.

### Statistical Analysis

The results of this study are presented as mean ± standard deviation (SD) for quantitative data. A *t*-test was used when the variance between two groups was the same (*F*-test ≤ 10%), otherwise, *t*′-test will be chosen. Frequencies and percentages were used to present qualitative data. A χ^2^ test or Wilcoxon test was used to compare differences between groups, and a univariate logistic regression model was developed to evaluate the statistical association between each factor and acPE. Forward stepwise regression was used to select ECG factors that were associated with acPE, and *p* < 0.05 was considered to be statistically significant. Furthermore, independent ECG predictors were analyzed using a multivariate logistic regression model. The test efficiency was evaluated using an area under the receiver operating characteristic curve (AUC). STATA 13.0 software was used for statistical analysis of the results (StataCorp, College Station, TX, USA).

## Results

### Study Sample

**Chart 1:**
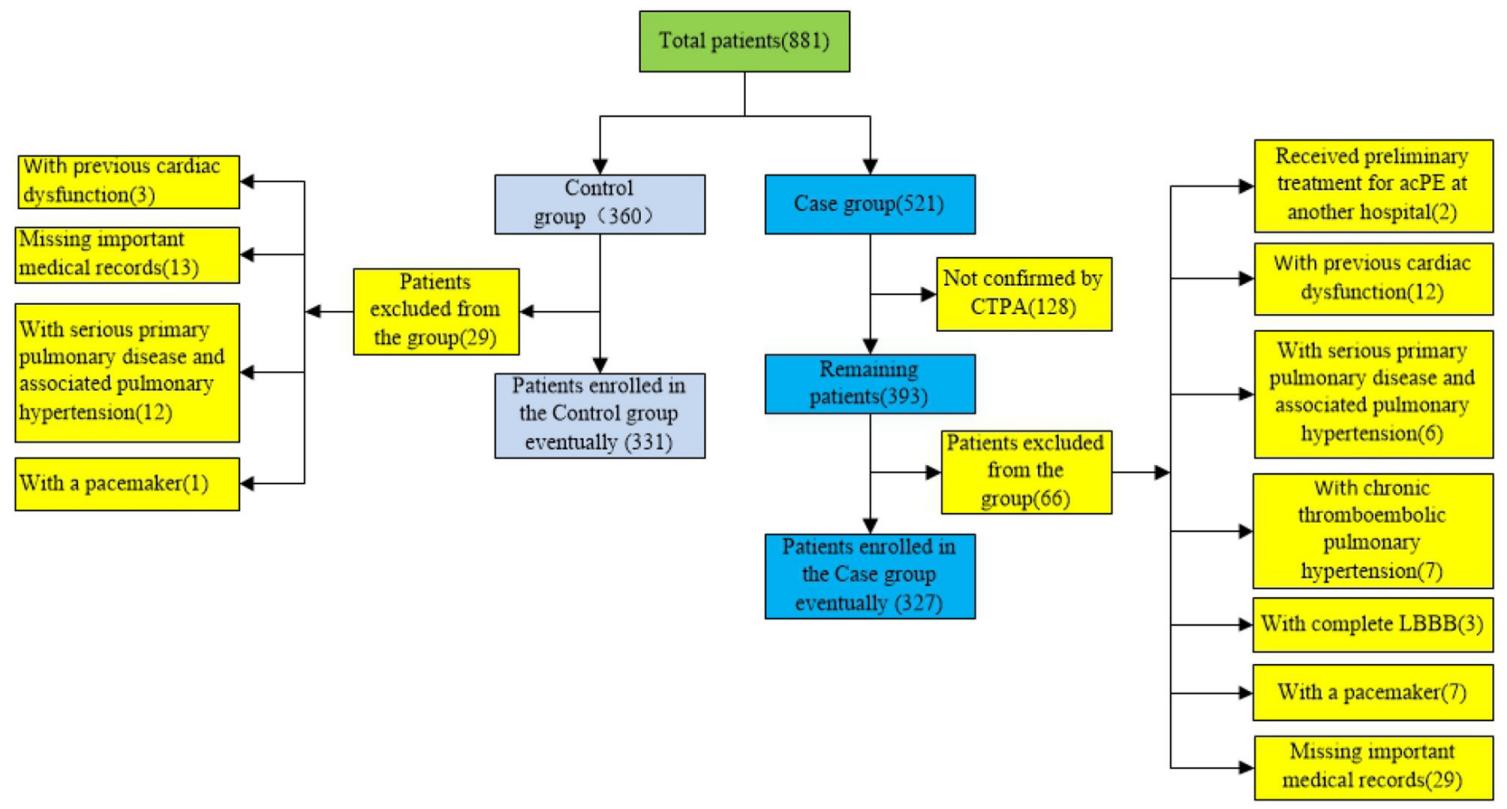
The flow chart of patient selection.

Of the 521 patients that had a diagnosis of acPE and were included in the disease cohort, 128 patient diagnoses were not confirmed by CTPA, so these patients were excluded from this cohort. Of the remaining 393 patients, 66 were excluded from the study due to 29 patients missing important medical records, 2 patients had received preliminary treatment for acPE at another hospital, 12 patients had experienced previous cardiac dysfunction, 6 patients had experienced serious primary pulmonary disease and associated pulmonary hypertension, 7 patients had chronic thromboembolic pulmonary hypertension, 3 patients had complete LBBB, and 7 patients had a pacemaker implanted prior to admission. Therefore, a total of 327 patients with acPE were included in this study. Among the patients hospitalized at the same time as the patients with acPE and had acPE ruled out *via* CTPA, a total of 360 patients were enrolled randomly in the control cohort. A total of 29 patients were excluded as 3 patients had previous cardiac dysfunction, 13 patients were missing important medical records, 12 patients had a serious primary pulmonary disease and associated pulmonary hypertension, and 1 patient had a pacemaker implanted prior to admission. Therefore, the control cohort included 331 patients in total. The flow chart patient selection is shown in Chart 1.

### Research Factors

In this study, 27 ECG signs and 12 clinical characteristics were analyzed and are presented in Table 1. The most common ECG signs were S1Q3T3 (case vs. control: 26.61 vs. 2.11%), complete or incomplete RBBB (case vs. control: 19.57 vs. 9.06%), and T-wave inversions in V1–V4 (case vs. control: 19.88 vs. 5.14%) based on the primary definition. The other ECG signs of right ventricular strain, including clockwise rotation (case vs. control: 35.17 vs. 11.18%), STE in leads V1–V3 (case vs. control: 10.43 vs. 2.72%), STE in lead AVR (case vs. control: 7.98 vs. 2.72%), qR/QR/Qr in lead V1 (case vs. control: 10.43 vs. 2.11%), P pulmonale (case vs. control: 14.37 vs. 2.11%), and atrial fibrillation (case vs. control: 10.70 vs. 2.72%), are presented in Table 2.

**Table 2:** The comparison of factors between control group and case group.

**Factors**	**-**	**Control group**	**Case group**	**Summation**	**Statistics**	***P***
AGE	Mean±SD	61.73 ± 14.71	65.25 ± 15.27	63.48 ± 15.08	*t* = 3.01	0.0027
SEX	0	109 (32.93%)	152 (46.48%)	261 (39.67%)	χ^2^ = 12.62	0.0004
	1	222 (67.07%)	175 (53.52%)	397 (60.33%)		
HISTORY1	0	328 (99.09%)	274 (83.79%)	602 (91.49%)	χ^2^ = 49.46	<0.0001
	1	3 (0.91%)	53 (16.21%)	56 (8.51%)		
HISTORY2	0	331 (100.00%)	249 (76.15%)	580 (88.15%)	χ^2^ = 89.57	<0.0001
	1	0 (0.00%)	78 (23.85%)	78 (11.85%)		
HISTORY3	0	228 (68.88%)	290 (88.69%)	518 (78.72%)	χ^2^ = 38.51	<0.0001
	1	103 (31.12%)	37 (11.31%)	140 (21.28%)		
HISTORY4	0	291 (87.92%)	282 (86.24%)	573 (87.08%)	χ^2^ = 0.41	0.5214
	1	40 (12.08%)	45 (13.76%)	85 (12.92%)		
HISTORY5	0	288 (87.01%)	208 (65.62%)	496 (76.54%)	χ^2^ = 41.28	<0.0001
	1	43 (12.99%)	109 (34.38%)	152 (23.46%)		
HISTORY6	0	329 (99.40%)	289 (88.38%)	618 (93.92%)	χ^2^ = 34.97	<0.0001
	1	2 (0.60%)	38 (11.62%)	40 (6.08%)		
HISTORY7	0	307 (92.75%)	194 (59.33%)	501 (76.14%)	χ^2^ = 101.14	<0.0001
	1	24 (7.25%)	133 (40.67%)	157 (23.86%)		
HISTORY8	0	328 (99.09%)	214 (65.44%)	542 (82.37%)	χ^2^ = 128.27	<0.0001
	1	3 (0.91%)	113 (34.56%)	116 (17.63%)		
PA	0	289 (87.31%)	162 (49.54%)	451 (68.54%)	χ^2^ = 108.83	<0.0001
	1	42 (12.69%)	165 (50.46%)	207 (31.46%)		
D2	Median (Q1–Q3)	0.40 (0.21 1.25)	6.23 (2.36 13.68)	1.66 (0.36 6.49)	z = 18.03	<0.0001
ECG1	0	296 (89.43%)	157 (48.01%)	453 (68.84%)	χ^2^ = 131.53	<0.0001
	1	35 (10.57%)	170 (51.99%)	205 (31.16%)		
ECG2	0	147 (44.41%)	44 (13.50%)	191 (29.07%)	z = 10.60	<0.0001
	1	95 (28.70%)	64 (19.63%)	159 (24.20%)		
	2	55 (16.62%)	118 (36.20%)	173 (26.33%)		
	3	34 (10.27%)	100 (30.67%)	134 (20.40%)		
ECG3	0	275 (83.08%)	153 (46.93%)	428 (65.14%)	z = 9.63	<0.0001
	1	19 (5.74%)	48 (14.72%)	67 (10.20%)		
	2	18 (5.44%)	58 (17.79%)	76 (11.57%)		
	3	19 (5.74%)	67 (20.55%)	86 (13.09%)		
ECG4	0	286 (86.40%)	171 (52.45%)	457 (69.56%)	z = 9.09	<0.0001
	1	11 (3.32%)	53 (16.26%)	64 (9.74%)		
	2	13 (3.93%)	40 (12.27%)	53 (8.07%)		
	3	21 (6.34%)	61 (18.71%)	82 (12.48%)		
	4	0 (0.00%)	1 (0.31%)	1 (0.15%)		
ECG5	0	328 (99.09%)	318 (97.55%)	646 (98.33%)	χ^2^ = 2.39	0.1221
	1	3 (0.91%)	8 (2.45%)	11 (1.67%)		
ECG6	Mean±SD	79.02 ± 19.94	90.48 ± 22.10	84.71 ± 21.79	*t* = 6.98	<0.0001
ECG7	0	318 (96.07%)	284 (87.12%)	602 (91.63%)	χ^2^ = 17.34	0.0002
	1	9 (2.72%)	26 (7.98%)	35 (5.33%)		
	2	4 (1.21%)	16 (4.91%)	20 (3.04%)		
ECG8	0	324 (97.89%)	292 (89.57%)	616 (93.76%)	χ^2^ = 19.41	<0.0001
	1	7 (2.11%)	34 (10.43%)	41 (6.24%)		
ECG9	0	299 (90.33%)	257 (78.83%)	556 (84.63%)	χ^2^ = 20.15	<0.0001
	1	9 (2.72%)	34 (10.43%)	43 (6.54%)		
	2	23 (6.95%)	35 (10.74%)	58 (8.83%)		
ECG10	0	328 (99.09%)	311 (95.40%)	639 (97.26%)	χ^2^ = 8.41	0.0037
	1	3 (0.91%)	15 (4.60%)	18 (2.74%)		
ECG11	0	326 (98.49%)	274 (84.31%)	600 (91.46%)	χ^2^ = 42.27	<0.0001
	1	5 (1.51%)	51 (15.69%)	56 (8.54%)		
ECG12	0	307 (92.75%)	285 (87.16%)	592 (89.97%)	χ^2^ = 5.70	0.0169
	1	24 (7.25%)	42 (12.84%)	66 (10.03%)		
ECG13	0	301 (90.94%)	263 (80.43%)	564 (85.71%)	z = 4.07	<0.0001
	1	7 (2.11%)	38 (11.62%)	45 (6.84%)		
	2	23 (6.95%)	26 (7.95%)	49 (7.45%)		
ECG14	0	314 (94.86%)	262 (80.12%)	576 (87.54%)	χ^2^ = 32.77	<0.0001
	1	17 (5.14%)	65 (19.88%)	82 (12.46%)		
ECG15	0	291 (87.92%)	210 (64.22%)	501 (76.14%)	χ^2^ = 50.84	<0.0001
	1	40 (12.08%)	117 (35.78%)	157 (23.86%)		
ECG16	0	318 (96.07%)	273 (83.74%)	591 (89.95%)	χ^2^ = 27.63	<0.0001
	1	13 (3.93%)	53 (16.26%)	66 (10.05%)		
ECG17	0	206 (62.24%)	90 (27.52%)	296 (44.98%)	z = 9.48	<0.0001
	1	13 (3.93%)	20 (6.12%)	33 (5.02%)		
	2	45 (13.60%)	81 (24.77%)	126 (19.15%)		
	3	60 (18.13%)	49 (14.98%)	109 (16.57%)		
	4	7 (2.11%)	87 (26.61%)	94 (14.29%)		
ECG18	0	294 (88.82%)	212 (64.83%)	506 (76.90%)	χ^2^ = 53.29	<0.0001
	1	37 (11.18%)	115 (35.17%)	152 (23.10%)		
ECG19	0	322 (97.28%)	292 (89.30%)	614 (93.31%)	χ^2^ = 16.81	<0.0001
	1	9 (2.72%)	35 (10.70%)	44 (6.69%)		
ECG20	0	323 (97.58%)	293 (89.60%)	616 (93.62%)	χ^2^ = 17.53	<0.0001
	1	8 (2.42%)	34 (10.40%)	42 (6.38%)		
ECG21	0	324 (97.89%)	280 (85.63%)	604 (91.79%)	χ^2^ = 32.81	<0.0001
	1	7 (2.11%)	47 (14.37%)	54 (8.21%)		
ECG22	0	313 (94.56%)	283 (86.54%)	596 (90.58%)	χ^2^ = 12.39	0.0004
	1	18 (5.44%)	44 (13.46%)	62 (9.42%)		
ECG23	0	317 (95.77%)	296 (90.52%)	613 (93.16%)	χ^2^ = 7.12	0.0076
	1	14 (4.23%)	31 (9.48%)	45 (6.84%)		
ECG24	0	318 (96.07%)	310 (94.80%)	628 (95.44%)	χ^2^ = 0.61	0.4344
	1	13 (3.93%)	17 (5.20%)	30 (4.56%)		
ECG25	0	277 (83.69%)	255 (77.98%)	532 (80.85%)	χ^2^ = 3.46	0.0630
	1	54 (16.31%)	72 (22.02%)	126 (19.15%)		
ECG26	0	302 (91.79%)	263 (80.43%)	565 (86.13%)	χ^2^ = 17.73	<0.0001
	1	27 (8.21%)	64 (19.57%)	91 (13.87%)		
ECG27	0	318 (96.07%)	255 (77.98%)	573 (87.08%)	χ^2^ = 47.86	<0.0001

**Figure 1:**
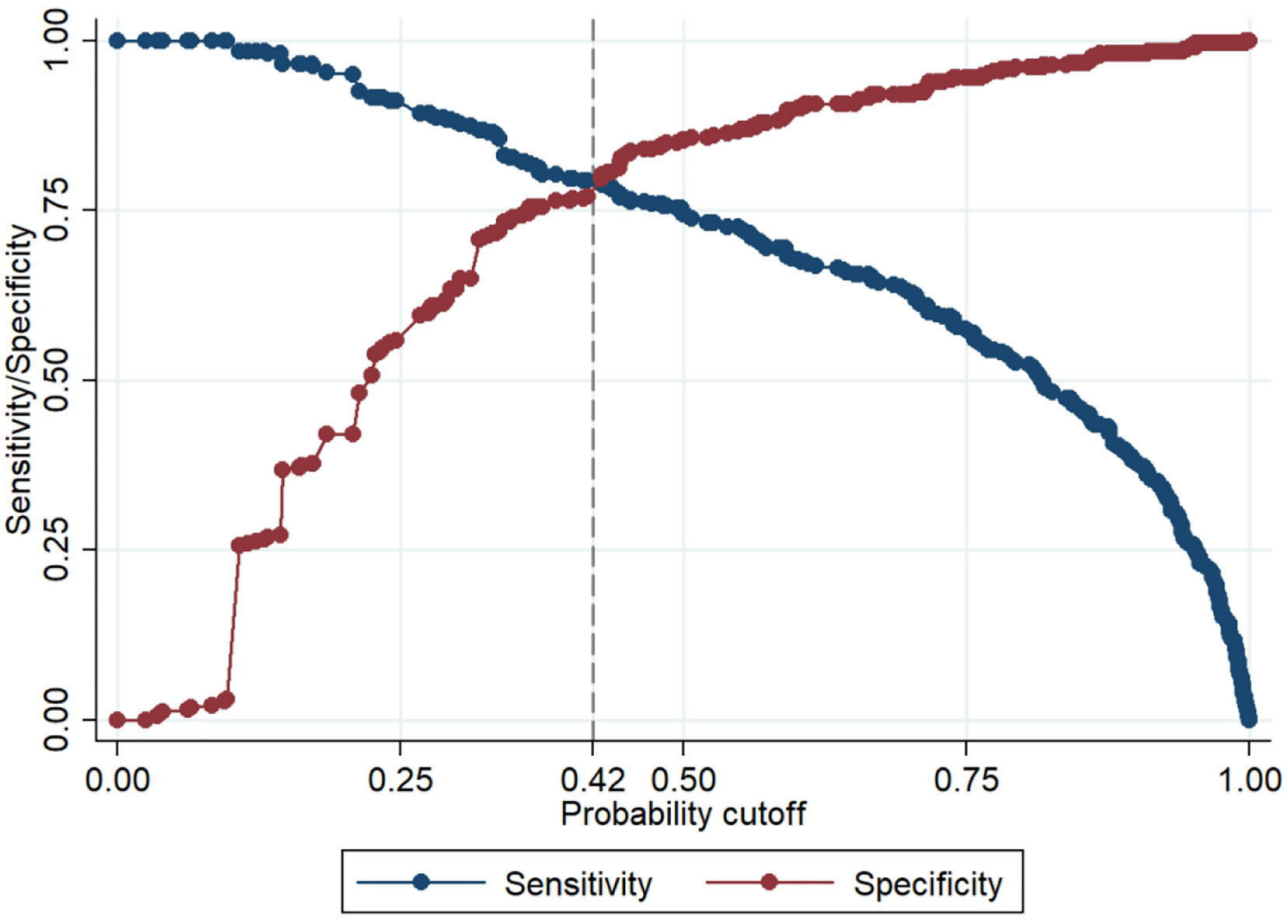
The sensitivity and specificity of our electrocardiogram (ECG) model intersected at the point 0.42, which means acute pulmonary embolism (acPE) should be considered when the probability is ≥ 0.42 and the corresponding score is −0.3228.


Table 3 indicates the univariate logistic regression model and the statistical associations between research factors and acPE. An ECG-predictive model was established using a forward stepwise regression and multivariate logistic regression model (Table 4). The following ECG signs were used in the model, namely, T-wave inversions in V1–V3, T-wave inversions in V1, T-wave inversions in V3, STE in lead AVR, STD in lead AVR, STE in leads V1–V3, STD in leads V1–V3, STE in inferior leads, supraventricular tachycardia, SI QIII TIII, atrial fibrillation, STE in lead V4–V6, and the R wave increase in lead AVR. Using this ECG model (Sichuan Provincial People's Hospital (SPPH)-ECG model), clinicians can evaluate ECG signs and the corresponding coefficients to calculate the probability of acPE. Figure 1 demonstrates that the sensitivity and specificity of SPPH-ECG model intersected at the point 0.42, indicating that acPE should be considered when the probability is ≥0.42 and the corresponding score is −0.3228.

**Table 3:** The result of univariate logistic regression analysis.

**Factors**	**B**	**SE**	**OR (95% CI)**	**χ^2^**	***P***	**Chi-squared test**	
						**χ^2^**	***P***
AGE	0.0157	0.0053	1.0159 (1.0054, 1.0264)	8.85	0.0029		
SEX	−0.5704	0.1612	0.5653 (0.4122, 0.7753)	12.53	0.0004		
HISTORY1	3.0505	0.5988	21.1261 (6.5330, 68.3162)	25.95	<0.0001		
HISTORY2	15.4876	226.5857	5323384 (0.0000, 3.95E199)	0.00	0.9455		
HISTORY3	−1.2643	0.2111	0.2824 (0.1867, 0.4272)	35.87	<0.0001		
TUMOR	0.1491	0.2328	1.1607 (0.7355, 1.8320)	0.41	0.5220		
HEARTRATE	1.2556	0.2018	3.5098 (2.3634, 5.2123)	38.72	<0.0001		
OTHER	4.0555	0.5914	57.7158 (18.1072, 183.9660)	47.02	<0.0001		
PA	1.9471	0.1988	7.0082 (4.7471, 10.3463)	95.97	<0.0001		
CF	2.1710	0.2400	8.7667 (5.4773, 14.0313)	81.84	<0.0001		
D2	0.4864	0.0466	1.6264 (1.4843, 1.7820)	108.80	<0.0001		
DVT	3.0741	0.7299	21.6298 (5.1728, 90.4439)	17.72	<0.0001		
AGE	0.0157	0.0053	1.0159 (1.0054, 1.0264)	8.85	0.0029		
SEX	−0.5704	0.1612	0.5653 (0.4122, 0.7753)	12.53	0.0004		
HISTORY1	3.0505	0.5988	21.1261 (6.5330, 68.3162)	25.95	<0.0001		
HISTORY2	15.4876	226.5857	5323384 (0.0000, 3.95E199)	0.00	0.9455		
HISTORY3	−1.2643	0.2111	0.2824 (0.1867, 0.4272)	35.87	<0.0001		
TUMOR	0.1491	0.2328	1.1607 (0.7355, 1.8320)	0.41	0.5220		
HEARTRATE	1.2556	0.2018	3.5098 (2.3634, 5.2123)	38.72	<0.0001		
OTHER	4.0555	0.5914	57.7158 (18.1072, 183.9660)	47.02	<0.0001		
PA	1.9471	0.1988	7.0082 (4.7471, 10.3463)	95.97	<0.0001		
CF	2.1710	0.2400	8.7667 (5.4773, 14.0313)	81.84	<0.0001		
D2	0.4864	0.0466	1.6264 (1.4843, 1.7820)	108.80	<0.0001		
DVT	3.0741	0.7299	21.6298 (5.1728, 90.4439)	17.72	<0.0001		
ECG1	2.2144	0.2102	9.1562 (6.0641, 13.8251)	110.95	<0.0001		
ECG2	0.8290	0.0829	2.2911 (1.9476, 2.6953)	100.04	<0.0001		
ECG3	0.7316	0.0865	2.0784 (1.7542, 2.4624)	71.50	<0.0001		
ECG4	0.6649	0.0881	1.9443 (1.6358, 2.3109)	56.91	<0.0001		
ECG5	1.0107	0.6814	2.7475 (0.7227, 10.4457)	2.20	0.1380		
ECG6	0.0266	0.0041	1.0269 (1.0187, 1.0352)	42.19	<0.0001		
ECG7_1 vs 0	1.1739	0.3953	3.2347 (1.4907, 7.0193)	8.82	0.0030	15.41	0.0005
ECG7_2 vs 0	1.4993	0.5649	4.4788 (1.4801, 13.5531)	7.04	0.0080		
ECG8	1.6843	0.4228	5.3887 (2.3528, 12.3419)	15.87	<0.0001		
ECG9_1 vs 0	1.4805	0.3844	4.3951 (2.0691, 9.3360)	14.83	0.0001	17.99	0.0001
ECG9_2 vs 0	0.5712	0.2816	1.7704 (1.0195, 3.0744)	4.12	0.0425		
ECG10	1.6625	0.6374	5.2725 (1.5118, 18.3879)	6.80	0.0091		
ECG11	2.4914	0.4750	12.0782 (4.7605, 30.6442)	27.51	<0.0001		
ECG12	0.6339	0.2688	1.8850 (1.1131, 3.1923)	5.56	0.0183		
ECG13	0.7255	0.1639	2.0658 (1.4983, 2.8483)	19.60	<0.0001		
ECG14	1.5222	0.2850	4.5823 (2.6213, 8.0104)	28.53	<0.0001		
ECG15	1.3995	0.2043	4.0532 (2.7157, 6.0494)	46.92	<0.0001		
ECG16	1.5579	0.3203	4.7488 (2.5348, 8.8967)	23.66	<0.0001		
ECG17	0.5229	0.0574	1.6869 (1.5075, 1.8877)	83.04	<0.0001		
ECG18	1.4610	0.2094	4.3103 (2.8594, 6.4973)	48.69	<0.0001		
ECG19	1.4559	0.3824	4.2883 (2.0268, 9.0734)	14.50	0.0001		
ECG20	1.5444	0.4011	4.6849 (2.1343, 10.2839)	14.82	0.0001		
ECG21	2.0502	0.4133	7.7694 (3.4563, 17.4647)	24.61	<0.0001		
ECG22	0.9946	0.2916	2.7036 (1.5267, 4.7876)	11.64	0.0006		
ECG23	0.8632	0.3320	2.3707 (1.2368, 4.5442)	6.76	0.0093		
ECG24	0.2937	0.3770	1.3414 (0.6407, 2.8084)	0.61	0.4359		
ECG25	0.3704	0.1998	1.4484 (0.9790, 2.1428)	3.44	0.0638		
ECG26	1.0013	0.2445	2.7219 (1.6856, 4.3952)	16.77	<0.0001		
ECG27	1.9322	0.3128	6.9045 (3.7399, 12.7470)	38.15	<0.0001		

**Table 4:** The result of multivariate logistic regression analysis.

**Factors**	**B**	**SE**	**OR (95% CI)**	**χ^2^**	***P***	**Chi-squared test**	
						**χ^2^**	***P***
Constant	−2.2342	0.2088	0.1071 (0.0711, 0.1612)	114.46	<0.0001		
ECG1	2.1780	0.4430	8.8286 (3.7055, 21.0348)	24.18	<0.0001		
ECG2	0.4475	0.1136	1.5645 (1.2521, 1.9547)	15.51	<0.0001		
ECG4	−0.4755	0.1900	0.6216 (0.4284, 0.9020)	6.27	0.0123		
ECG7_1 vs 0	1.2680	0.5716	3.5538 (1.1592, 10.8953)	4.92	0.0265	14.17	0.0008
ECG7_2 vs 0	2.0232	0.6560	7.5625 (2.0906, 27.3557)	9.51	0.0020		
ECG9_1 vs 0	0.5832	0.4836	1.7918 (0.6945, 4.6228)	1.45	0.2278	9.49	0.0087
ECG9_2 vs 0	−1.0803	0.4092	0.3395 (0.1522, 0.7570)	6.97	0.0083		
ECG11	2.2346	0.5497	9.3423 (3.1807, 27.4399)	16.52	<0.0001		
ECG15	0.9330	0.2634	2.5421 (1.5170, 4.2599)	12.55	0.0004		
ECG17	0.3325	0.0747	1.3944 (1.2044, 1.6143)	19.80	<0.0001		
ECG19	1.5452	0.4824	4.6887 (1.8215, 12.0696)	10.26	0.0014		
ECG21	1.5669	0.4987	4.7916 (1.8029, 12.7349)	9.87	0.0017		
ECG22	1.0471	0.3699	2.8493 (1.3800, 5.8829)	8.01	0.0046		
ECG27	1.2782	0.4088	3.5902 (1.6112, 8.0001)	9.78	0.0018		

*The SPPH-ECG model: $\pi \left(Y=1\right)=\frac{1}{1+\exp\left(\mathrm{Score}\right)}$*.

*Score = −2.3242+2.1780 × ECG1+0.4475 × ECG2+……+1.2782 × ECG27*.

$$\begin{array}{l} \text{The SPPH}-\text{ECG model }:\;\pi \left(Y=1\right)=\frac{1}{1+\exp\left(Score\right)}\\ Score=-2.3242+2.1780\times ECG1+0.4475\times ECG2+\ldots \ldots \\ \;+1.2782\times ECG27 \end{array}$$

**Figure 2:**
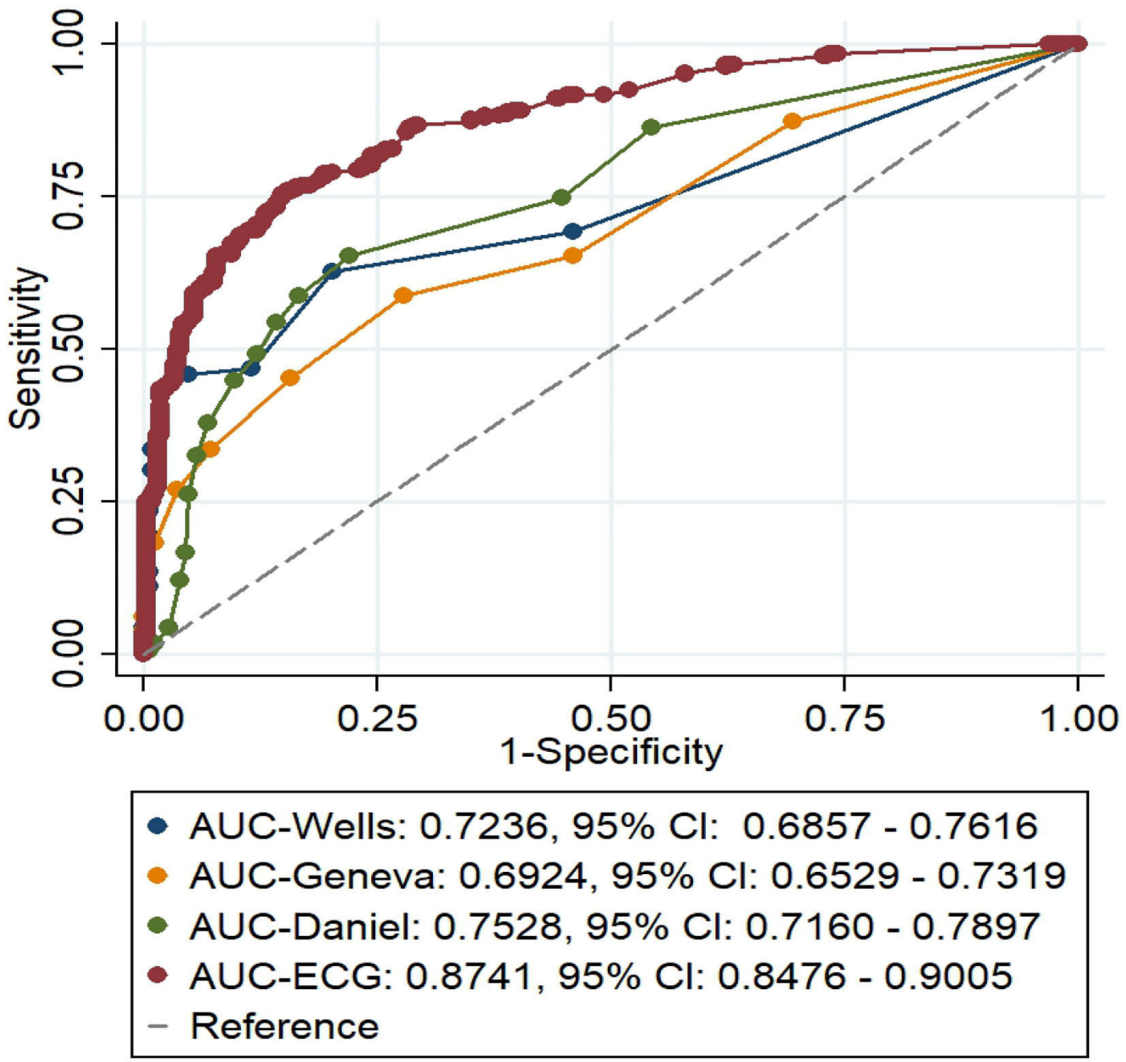
The comparison of area under the receiver operating characteristic curve (AUC) of the SPPH-ECG model, Daniel-ECG score, the Wells score, and Geneva score.

Additionally, the diagnostic values of this ECG model, the Daniel-ECG score,Wells score simplified, and Geneva score revised, simplified were compared using AUC (Figure 2). The model established in this study had a superior AUC (0.8741) compared with the other scoring systems investigated. In the validation cohort, the Daniel-ECG score, Wells score, and Geneva score exhibited favorable specificity and a positive predictive value and exhibited poor sensitivity and a negative predictive value. The SPPH-ECG model sensitivity (79.08%), specificity (79.76%), positive predictive value (79.32%), and negative predictive value (79.52%) were preferable to the other scoring systems analyzed. The accuracy of the SPPH-ECG model (79.42%) was also superior to the other three scoring systems (Daniel 60.79%, Wells simplified 56.69%, and Geneva revised, simplified 53.34%), as presented in Table 5.

**Table 5:** The comparison of the sensitivity, specificity, accuracy, and positive and negative predictive values of the four models.

**Indicators**	**Wells simplified**	**Geneva revised, simplified**	**Daniel**	**SPHH-ECG**
Sensitivity	13.46%	6.12%	25.99%	79.08%
Specificity	99.40%	100.00%	95.17%	79.76%
Positive predictive value	95.65%	100.00%	84.16%	79.32%
Negative predictive value	53.76%	51.89%	56.55%	79.52%
Accuracy	56.69%	53.34%	60.79%	79.42%

## Discussion

Following the development of an ECG scoring system by Daniel et al. in 2001, there has been an increasing volume of research that has focused on ECG-specific changes in patients with acPE. Furthermore, a number of previously unreported ECG characteristics are gaining attention for their potential value in aiding the diagnosis of acPE. The current ESC guidelines for the diagnosis of acPE suggest that ECG changes such as inversion of T waves in leads V1–V4, a QR pattern in V1, an S1Q3T3 pattern, and an incomplete or complete RBBB are usually identified in more severe cases of PE (2, 24). These guidelines also state that in milder cases of the disease the only ECG abnormality may be sinus tachycardia, which is present in 40% of patients with acPE. Atrial arrhythmias, and most frequently atrial fibrillation, may also be associated with acPE (2). Therefore, it is necessary to develop a new scoring system using the study off all associated ECG changes in patients with this disease. This study analyzed a number of ECG indicators for acPE and developed a novel scoring system, in which a number of novel ECG signs, including STE or STD in lead AVR, STE, or STD in leads V1–V3, the Q wave in the inferior leads (Q ≥ 0.15 mV), atrial fibrillation, P pulmonale, premature atrial contraction (PAC), and R/S ≥ 1 in lead AVR, were included.

There are a number of different studies that have demonstrated the occurrence of STE in leads V1–V3 in patients with acPE (25–30). A number of different explanations have been proposed for the cause of right precordial lead STE, which is thought to be due to the right ventricle (RV) transmural ischemia in the majority of cases (11). The underlying mechanism for the development of RV transmural ischemia in acPE is not well understood, and a number of explanations as to why this occurs have been previously proposed. During RV dilation and failure, the RV may be unable to generate enough systolic pressure to overcome the acute increase in afterload, leading to increased RV oxygen demand and a significant reduction in pulmonary perfusion. This, together with a leftward shift of the interventricular septum, will reduce the left ventricular preload and, subsequently, the cardiac output and coronary flow, which, in addition to the ensuing hypoxia, can cause severe RV transmural ischemia, leading to STE in leads V1–V3/V4 (31–33). Q wave in the inferior leads and QR or qR complexes in lead V1 can be explained by the posterior displacement of the initial depolarization vector, normally giving rise to the r wave in lead V1 and the q wave in the anterolateral leads, directed left to right and anteriorly and rotated away from lead V1, because the dilated RV pushes backward and compresses the left ventricle (15, 34, 35). Acute RV failure, tricuspid valve insufficiency, and neurohormonal activation may lead to atrial arrhythmias and most frequently atrial fibrillation, which may be associated with acPE (2). In a recent large study of 975 patients that was conducted by Kukla et al., atrial fibrillation was observed in 231 (24%) patients with acPE (36). The presence of RV enlargement, heart transposition, and right axis deviation may explain the R/S ≥ 1 in lead AVR.

PE is the third most common cause of death from cardiovascular disease in the USA. Despite the high prevalence of this disease, PE is difficult to diagnose, with only 43–53 patients/100,000 being accurately diagnosed, and up to 70% of clinically unsuspected PE diagnosed at autopsy (37–39). Rapid diagnosis of this life-threatening disease is important, and rapid diagnostic methods with high sensitivity are urgently required. The superior overall diagnostic accuracy of the SPPH-ECG model to predict the pre-test probability of acPE compared to other scoring methods is based on the system's superior sensitivity, negative predictive value, and test accuracy. However, the specificity of the SPPH-ECG model was inferior to those of the other three scoring systems analyzed. An ECG, clinical history, and physical examination can be rapidly performed within the emergency department.

Therefore, the SPPH-ECG model combined with the Geneva score may be used to diagnose patients with acPE rapidly and accurately. This study has a number of limitations. It is retrospective, and larger multicenter prospective studies are required to confirm the diagnostic value of the SPPH-ECG model. The inadequacy of the SPPH-ECG model for application in patients with LBBB, previous cardiac dysfunction, serious primary pulmonary disease (i.e., serious pulmonary emphysema or pneumonitis), and persistent pacemaker rhythm, and the underrepresentation of patients with peripheral PE in this study are also important limitations to note. Furthermore, it did not analyze the ECG changes of right-sided chest leads in patients with acPE.

Increasing evidence suggests that analysis of ECG results may serve a valuable diagnostic role in patients with acPE, particularly when modern technology may not be readily accessible. Many patients with acPE exhibit no representative ECG signs when the Daniel et al. scoring system is applied, so this limits the application of an ECG in the diagnosis of acPE. However, due to its very high sensitivity and negative predictive value, SPPH-ECG model may serve a role in ruling out acPE in patients. In conclusion, SPPH-ECG model in combination with the Geneva score may help clinicians diagnose acPE more rapidly and accurately than when using some scoring systems currently available.

## Data Availability Statement

The original contributions presented in the study are included in the article/supplementary material, further inquiries can be directed to the corresponding author.

## Ethics Statement

The studies involving human participants were reviewed and approved by Medical Ethics Committee, Sichuan Academy of Medical Sciences, Sichuan Provincial People's Hospital. Written informed consent was not required for this study, in accordance with the local legislation and institutional requirements.

## Author Contributions

X-FS: conceptualization, methodology, and writing. XL: conceptualization, methodology, review, and editing. NF: data curation and formal analysis. X-MY: data curation. J-MS and Q-HP: software. All authors contributed to the article and approved the submitted version.

## Conflict of Interest

The authors declare that the research was conducted in the absence of any commercial or financial relationships that could be construed as a potential conflict of interest.

## Publisher's Note

All claims expressed in this article are solely those of the authors and do not necessarily represent those of their affiliated organizations, or those of the publisher, the editors and the reviewers. Any product that may be evaluated in this article, or claim that may be made by its manufacturer, is not guaranteed or endorsed by the publisher.
